# Survival following gamma knife radiosurgery for brain metastasis from breast cancer

**DOI:** 10.1186/1748-717X-8-131

**Published:** 2013-05-29

**Authors:** Jerry J Jaboin, Daniel J Ferraro, Todd A DeWees, Keith M Rich, Michael R Chicoine, Joshua L Dowling, David B Mansur, Robert E Drzymala, Joseph R Simpson, William J Magnuson, Anushka H Patel, Imran Zoberi

**Affiliations:** 1 Department of Radiation Oncology, Washington University School of Medicine, St. Louis, MO, USA; 2 Department of Neurological Surgery, Washington University School of Medicine, St. Louis, MO, USA; 3 Department of Radiation Oncology, Case Western Reserve University, Cleveland, USA; 4 Department of Human Oncology, University of Wisconsin School of Medicine and Public Health, Madison, WI, USA; 5 Arizona Oncology Services, Phoenix, AZ, USA

**Keywords:** Gamma knife, Stereotactic radiosurgery, GKS, Breast cancer, Brain metastasis

## Abstract

**Background:**

Breast cancer is the second most common cause of brain metastases in the United States. Although breast cancer induced brain metastases represent an incurable condition, some patients experience prolonged survival. In this retrospective study, we examine a cohort of patients with brain metastases from breast cancer treated with Gamma Knife stereotactic radiosurgery to identify factors that predict better outcomes.

**Methods:**

A retrospective database of 100 patients treated for brain metastases due to breast cancer via Gamma Knife radiosurgery (GKS) from July 1998 through March 2009 was reviewed. Patients who received radiosurgery as sole treatment, as a planned boost after whole brain radiotherapy or surgical resection, or as salvage after prior whole brain radiation therapy (WBRT) or surgical resection were included. Prognostic factors identified to be significant for survival in previous brain metastasis studies were analyzed for significance by univariate and multivariate Cox analysis.

**Results:**

Overall, the median brain progression-free survival time was 7.1 months and the median survival time was 12.3 months. No prognostic variables were significant for brain progression-free survival. For patients treated with a planned GKS after WBRT, GKS as sole treatment, GKS salvage after WBRT, GKS boost after surgery, or GKS for surgical salvage the median survival times (MSTs) were as follows: 12.2 months, 12.4 months, 9.5 months, 27.6 months and 33.4 months respectively. Differences between the groups were not significant (*p* = 0.06); however, GKS boost after surgery and GKS for salvage after surgery did have a trend toward better overall survival.

The MST for patients of age <65 years was 14.5 months, compared to age ≥65 which was 7.7 months (*p* = 0.06) and remained a significant prognostic factor for overall survival on multivariate analysis. The MST for patients with a single lesion was 16.9 months, not significantly different than the MST of 14.5 months for patients with 2–3 lesions. However patients with >3 lesions had a MST of 5.9 months, which was significantly worse. Breast cancer subtype as approximated by biomarkers and KPS were not significant predictors of overall survival and stage at initial diagnosis was inversely associated with survival.

**Conclusion:**

Stereotactic radiosurgery offers good local control and prolonged survival in selected patients. Age and number of lesions are strong predictors of overall survival.

## Introduction

Breast cancer is the most common cancer among women, and the second most common source of brain metastases in the United States. While major advances in the treatment of breast cancer have occurred in the past two decades, a significant number of women will continue to succumb to the disease after metastatic spread. Approximately 10–15% of women diagnosed with breast cancer will develop symptomatic brain metastases, and approximately 30% of breast cancer patients will have brain disease at autopsy. Young age, estrogen receptor negative status, and HER2 overexpression have all been associated with increased risk for the development of breast cancer CNS metastases [1,2].

Supportive care and whole brain radiation therapy (WBRT) have a major role in the management of brain metastasis; however, the survival of most patients with brain metastasis remains limited. In this population the Radiation Therapy Oncology Group (RTOG) has identified age, performance status, control of the primary tumor, and presence of extracranial disease as prognostic factors for survival [3,4] and created a recursive partitioning analysis (RPA) to predict survival using these factors. Unfortunately this model demonstrated that even the most favorable patients had a median survival of only 7.1 months [3]. The Breast Specific Graded Prognostic Assessment (BS-GPA) showed patients with CNS metastases due to breast cancer to have a median survival of 13.8 months, with the most favorable group having a median survival of 25.3 months [5]. These median survival times were much longer than that predicted by the RTOG RPA and suggest that patients with CNS metastases due to breast cancer survive longer than patients with CNS metastases as a whole. The Breast Specific Graded Prognostic Assessment is defined in *Sperduto et al. 2012* and utilizes Karnofsky Performance Status (KPS), Genetic subtype, and Age to risk stratify patients into 4 groups predictive for median survival [5].

In an attempt to capitalize on these results, some patients with good performance status, limited extracranial disease, and few brain lesions have been treated more aggressively with stereotactic radiosurgery (SRS). Originally developed by neurosurgeon Lars Leksell [6], stereotactic radiosurgery delivers a single high dose of ionizing radiation to a small volume in a highly accurate manner. Currently SRS is most often performed via a linear accelerator or a Gamma Knife (Elekta Instruments, Norcross, GA). Defining optimal situations for SRS has proved difficult with practitioners using SRS as a planned boost following WBRT [7], a planned boost to a surgical bed [8,9], sole treatment [10], or as a salvage intervention [11]. Here we report our experience treating CNS breast metastasis via Gamma Knife Radiosurgery.

## Methods

### Patient selection

Patients with a prior pathologic diagnosis of breast cancer treated with Gamma Knife radiosurgery (GKS) for breast cancer related brain metastases at the Siteman Cancer Center at Barnes-Jewish Hospital/Washington University Medical Center (BJH/WUMC) between July 1998 and March 2009 were retrospectively reviewed. Our Gamma Knife center is a shared community resource utilized by qualified neurosurgeons and radiation oncologists from the Greater Saint Louis area. This review was restricted to patients treated by neurosurgical and radiation oncology faculty of Washington University School of Medicine (WUSM). There were no other exclusions. The primary endpoint of the study was overall survival with the rate of disease recurrence in the brain being a secondary endpoint. Patients who had previous whole brain radiotherapy or prior surgical resection were included. Patients who underwent surgical resection were medically stable at the time of surgery and believed to have life expectancies >6 months. Patients with evidence of residual disease in the surgical field on the first postoperative MRI were classified as having a sub-total resection and not recurrent disease. Breast cancer subtype for invasive cancers was approximated using estrogen receptor (ER), progesterone receptor (PR), and Human Epidermal growth factor Receptor 2 (HER2/neu) status [8]. Patients were followed serially in both the Neurosurgical and Radiation Oncology clinics. Patients were assessed for KPS and neurological symptoms. Patients were classified using the RTOG RPA, GPA, and BS-GPA classification system. Washington University School of Medicine Human Research Protection Office reviewed and approved this study (Protocol# 201106242).

### Radiosurgical technique

Stereotactic radiosurgery was performed at the Gamma Knife of Saint Louis facility at BJH/WUMC. We categorized radiosurgery by its intent as Sole Treatment, Planned Boost after Whole Brain Radiation, Planned Boost after Surgery or Salvage Treatment. We defined Sole Treatment as being GKS administered without any prior radiation therapy, surgery, or chemotherapy specifically intended to treat brain metastasis. A Planned Boost occurred within two months of the completion of either WBRT or surgery. Salvage treatment occurred in the setting of prior radiation therapy, surgery, or chemotherapy for brain metastasis. All patients were assigned to a treatment intent category based on their clinical situation at their initial GKS treatment. Any subsequent GKS treatments for a particular patient were classified as an in-brain recurrence and not as independent cases.

All patients underwent stereotactic radiosurgery with the Gamma Knife (Elekta, Norcross, GA). From June 1998 to August 2002 this was a Model B while from August 2002 to April 2008 a Model C and afterwards a Perfexion. All patients had intravenous access placed. A Leksell frame was placed under local anesthesia often with a low, intravenous dose of an anxiolytic. In the vast majority of cases a computed tomography scan without contrast and a contrast-enhanced magnetic resonance image were obtained and images transferred to the GKS treatment planning computer. The target(s) were contoured and a radiosurgery plan was developed. In general, dose was selected based on the recommendations from the SRS dose escalation trial RTOG 90–05 [12]; tumor size, location, and history of prior radiation therapy were considered in selecting the prescription dose. The doses ranged from 10 to 24 Gy, The prescription dose was generally prescribed to the 50% isodose line, which followed the MR contrast enhanced tumor margin. Quality assurance consisting of verification of patient name from all imaging, patient orientation, MR fiducial alignment, patient identity (patient name and birth date by all members of the treatment team), and treatment specifications at the Gamma Knife treatment console was done in all cases.

The medical record was retrospectively reviewed to determine pretreatment patient and tumor characteristics and the dates of time-to-event endpoints. The recurrence of treated disease, progression of known disease, or the development of new brain metastasis was scored as a failure for brain recurrence. Death from any cause was a failure for overall survival. All times were measured from the date of radiosurgery.

### Statistical analysis

Standard measures of central tendency and dispersal were used to characterize patient and tumor parameters. Survival time and time to CNS recurrence were calculated from the date of GKS. The Kaplan-Meier method was used to describe time to brain recurrence and overall survival. The log-rank test was used to compare these endpoints in univariate analysis while the Cox Proportional Hazards method was used to analyze the effects of multiple potential independent predictors on overall survival. Based on published literature, we analyzed several known favorable predictors including: controlled extracranial disease, number of brain metastases, no prior whole brain radiotherapy, smaller total radiosurgery volume, HER2/neu overexpression, younger age, and good performance status. Suspected predictive factors including treatment intent and number of brain metastasis were entered using stepwise deletion. Statistical tests were performed using SAS version 9.2 (SAS Institute Inc., Cary, NC). All statistical tests were two-sided with *p* values < 0.05 deemed significant.

## Results

### Patient characteristics

One hundred consecutive breast cancer patients receiving Gamma Knife stereotactic radiosurgery (GKS) for brain metastasis treated between July 1998 and March 2009 were reviewed. All patients were female. The mean time from the diagnosis of breast cancer to the detection of brain metastasis was 48 months (median: 30.2 months), while the mean time from diagnosis of breast cancer to radiosurgery was 55 months (median: 37.4 months). The mean time from diagnosis of brain metastases to GKS was 7 months (median: 3.4 months). For GKS treatment, the mean follow-up time was 18.3 months (median: 12.3 months). Complete information regarding TNM (Tumor, Node and Metastasis) staging was available in 87 patients while the remaining 13 were only known to be M0 or M1 at diagnosis. In our cohort, 13% were Stage I, 27% Stage II, 34% Stage III and 13% Stage IV with 85 patients having had M0 disease at the time of diagnosis. Detailed patient, tumor and treatment characteristics are summarized in Table 1.

**Table 1:** Patient characteristics

**Characteristics**	**No. of patients (n = 100)**
Age at GKS, mean (sd), y	51.8 (10.2)
M0 at diagnosis	85
Breast Cancer Subtype	
Luminal A	15
Luminal B	20
HER2	33
Basal	27
Unknown	5
KPS score	
70–80	73
90–100	27
Extracranial metastasis at time of GKS	71
RTOG RPA Class 1	24
RTOG RPA Class 2	76
Prior WBRT	64
Number of Brain Metastasis Treated	
1	48
2	21
3	17
4	10
> = 5	4
Intent of Radiosurgery	
Boost after Surgery	9
Boost after WBRT	25
Sole Treatment	26
Salvage after Surgery	3
Salvage after WBRT	37
Volume of Largest Lesion, mean (sd), mL	3.86 (4)

Abbreviations: *GKS* Gamma Knife radiosurgery, *ER* estrogen receptor, *H2N* her-2/neu gene, *KPS* Karnofsky Performance Status, *RPA* Recursive partitioning analysis, *RTOG* Radiation therapy oncology group, *WBRT* Whole brain radiation therapy.

### Radiosurgical parameters

All evaluated patients were treated with GKS as described in the Methods section. The median tumor volume was 3.50 cm^3^ (range, 0.10–38.71). The median treatment volume was 3.69 cm^3^ (range, 0.12–141.4). The median number of treatment shots was 8 (range, 1–35). The median number of lesions was 2 (range, 1–20). The median number of treated lesions was 2 (range, 1–12). The median GKS prescription dose was 18 Gy to the 50% isodose line (range, 10–24 Gy).

### General patient survival outcomes

Fifty-three patients had a CNS failure, however not all received subsequent CNS treatment: 15 had subsequent GKS, 1 had surgery, 9 had subsequent WBRT, 3 had both WBRT and GKS. The median overall survival time for our patient cohort was 12.3 months from the time of GKS treatment. At our last update, 88 of our 100 patients were deceased. The median progression-free survival time was 7.1 months.

### Patient outcomes based on treatment intent

**Figure 1:**
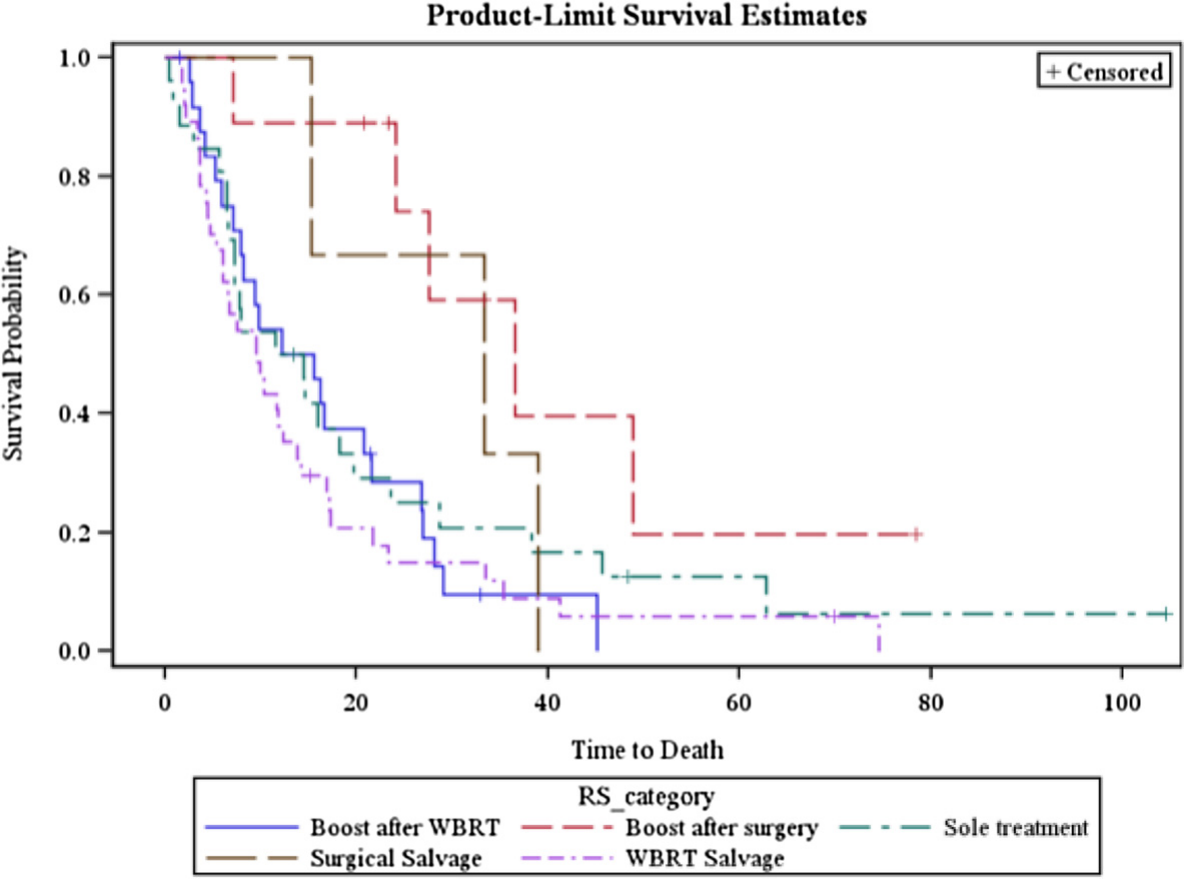
Overall survival of patients treated by GKS stratified by type of treatment.

The nine patients treated with a GKS boost after surgery had a median survival time (MST) of 27.6 months. The three patients undergoing GKS for surgical salvage had a MST of 33.4 months. The 25 patients treated with a planned GKS boost after whole brain radiation therapy (WBRT) had a MST of 12.2 months. The 26 patients who had GKS as sole treatment had a MST of 12.4 months, and the 37 patients who had GKS for salvage after WBRT had a MST of 9.5 months. There was no statistical significance between the groups in terms of overall survival. Grouping the overall cohort into those receiving GKS as salvage therapy, sole therapy, or as a planned boost also resulted in non-significantly different survival curves (Figure 1).

### Patient outcomes based on known prognostic parameters

**Figure 2:**
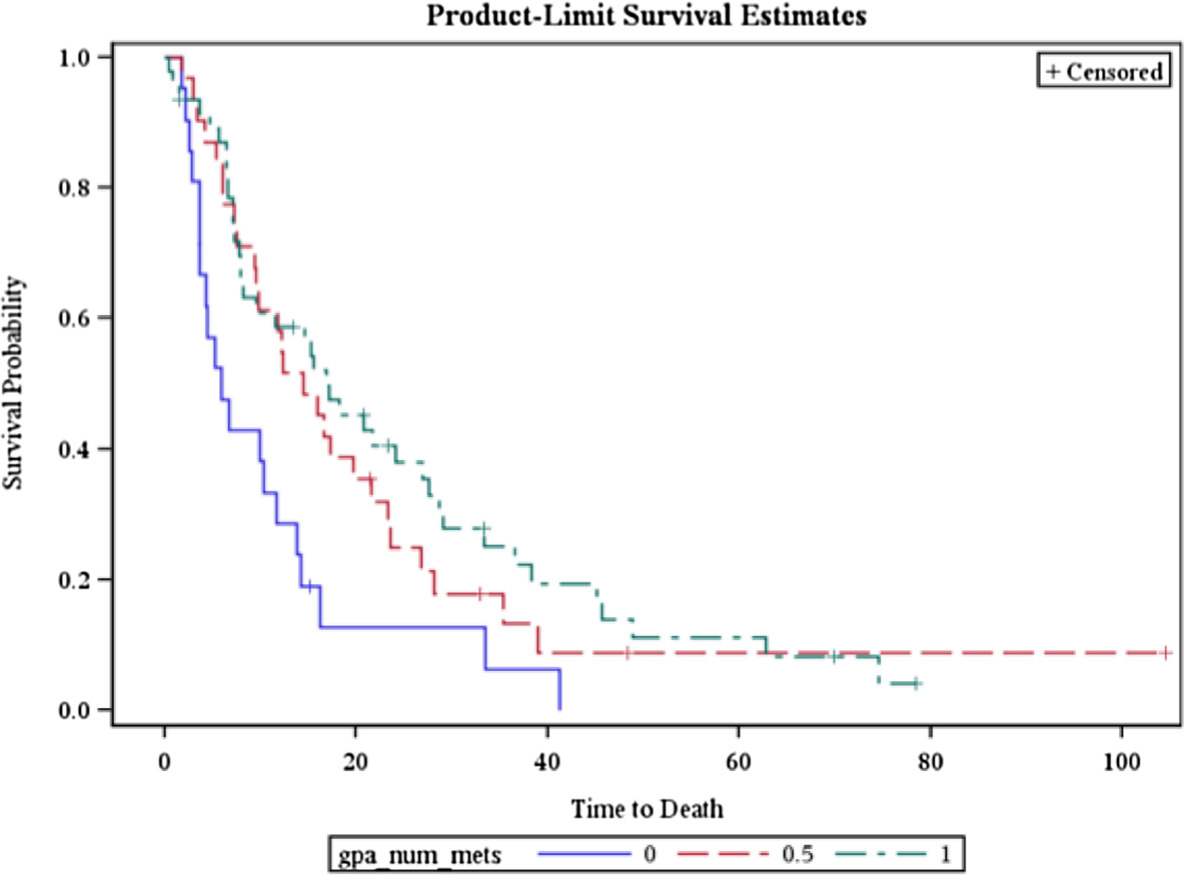
**Overall survival of patients treated by GKS stratified by number of lesions.** 0= > 3, 0.5 = 2–3, 1 = 1.

There was a significant difference in outcomes based on the number of brain lesions (Chi square: 10.06, *p* = 0.01), as shown in Figure 2. Patients with a single treated lesion had a MST of 16.9 months. To analyze the effect of multiple lesions, we compared patients with >3 lesion (MST: 5.9 months) and patients with 2–3 lesions (MST: 14.5 months) to patients with a single lesion. This analysis demonstrated that patients with >3 lesions have a significantly lower overall survival (HR = 2.42, *p* < 0.01), while patients with 2–3 lesions show no significant difference in survival time (HR = 1.91, *p* = 0.49) as compared to the patients with a single lesion.

**Figure 3:**
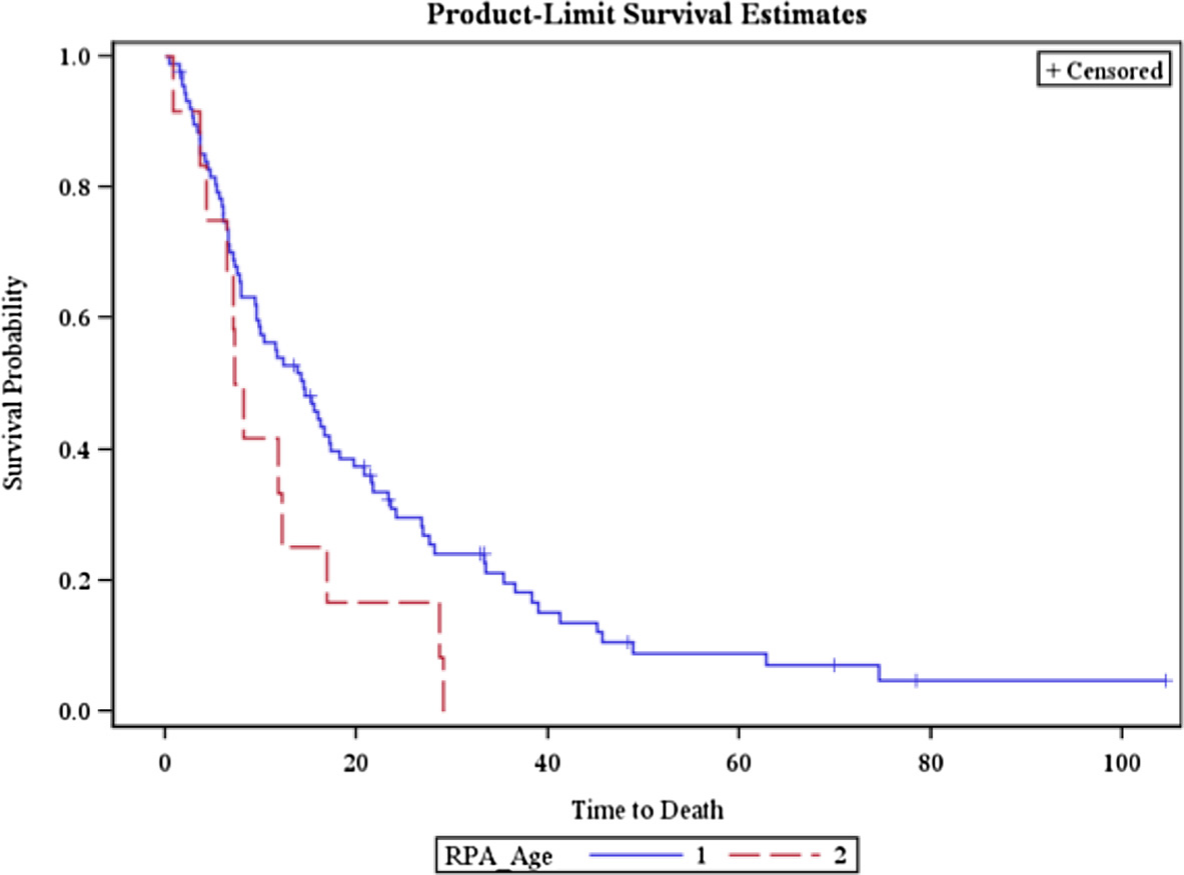
**Overall survival of patients treated by GKS stratified by age.** 1 = <65, 2= >/=65.

The MST for patients with age <65 years was 14.5 months, and for patients with age ≥65 was 7.7 months (Figure 3). While the data was not statistically significant, there was a strong trend towards a difference in survival (HR = 0.61, Chi square: 3.41, *p* = 0.06) in favor of lower age, which did become significant on multivariate analysis (HR = 0.28, Chi square 6.42, p = 0.01).

Patients were categorized into five tumor subtypes based on surface receptor expression: luminal A, luminal B, HER2/neu, basal, and unknown. The MST for luminal A was 7.2 months, luminal B was 17.8 months, basal was 8.1 months, HER2/neu was 21.7 months and unknown was 7.8 months. Overall there was no difference seen between these subtypes on univariate analysis (Chi square: 6.51, *p* = 0.16).

Documented CNS failure occurred in 51.4% of the patients. CNS failure was associated with a significant increase in MST, where patients with CNS failure had a MST of 14.5 months and patients without CNS failure had a MST of 6.54 months. There was a statistically significant overall survival advantage seen for patients who had documented CNS failure, as compared to those who did not (HR = 0.56, *p =* 0.01). Multivariate analysis demonstrated CNS failure to predict longer MST (Chi Square: 4.79, *p* = 0.03).

Stage at initial presentation predicted lower MSTs for stage I after GKS for CNS disease. The MST for stage I was 5.9 months, stage II was 14.4 months, stage III was 13.0 months, and stage IV was 9.8 months (Chi square: 9.88, *p* = 0.02). On multivariate analysis, stage remained a significant predictor of overall survival (Chi Square: 8.67, *p* = 0.03).

### Patient outcomes based on known prognostic assessments

**Figure 4:**
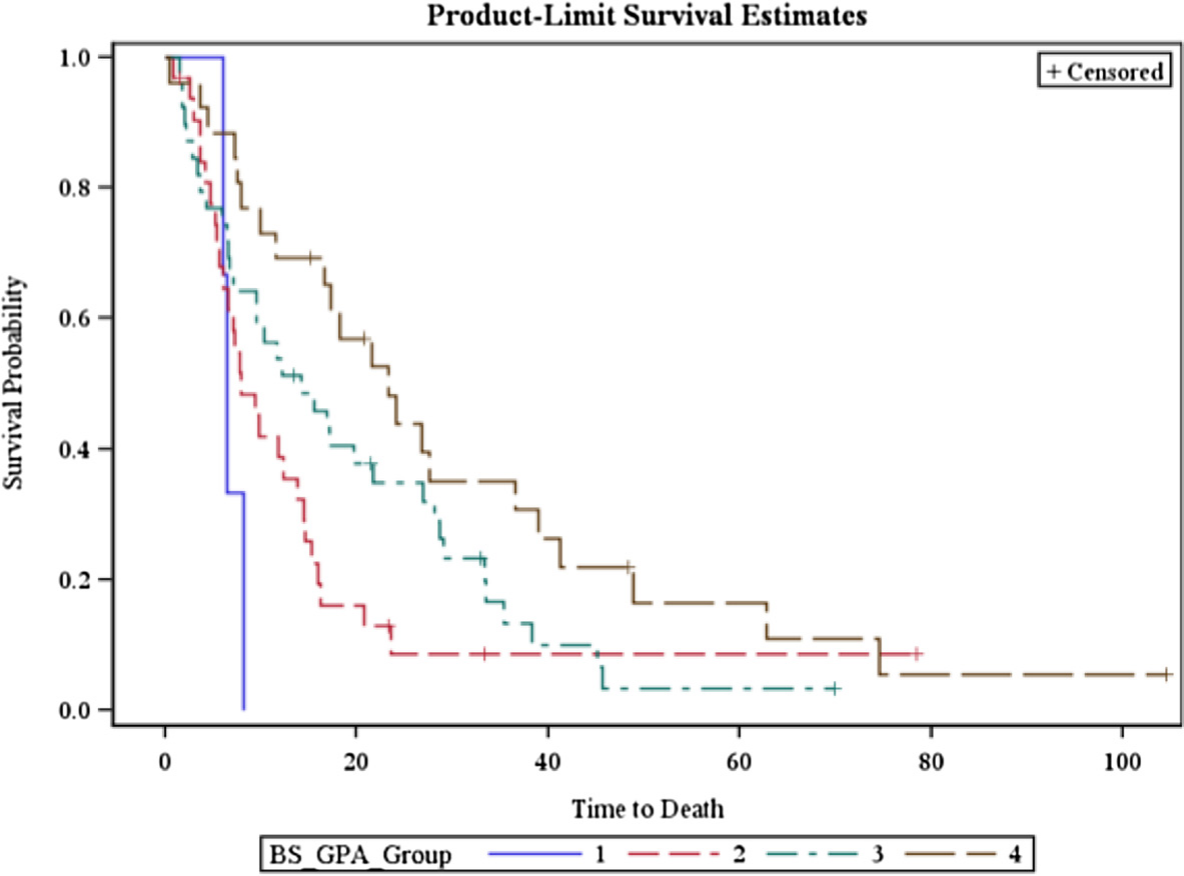
Overall survival of patients treated by GKS stratified by Breast Specific GPA.

The BS-GPA was predictive of survival outcomes (Chi square: 11.36, *p* = 0.01, Figure 4). For patients with a BS-GPA of 0–1 (Group 1), the MST was 6.4 months. For the others: BS-GPA of 1.5–2.0 (Group 2) had a MST of 7.9 months, the BS-GPA of 2.5–3.0 (Group 3) had a MST of 13.3 months, and those with a BS-GPA of 3.5–4.0 (Group 4) had a MST of 21.1 months.

## Discussion

Randomized trials have tested SRS with and without WBRT [7,13]. These studies demonstrated median survival times between 6.5 to 8.0 months. It is difficult to apply these outcomes to breast cancer patients, as they comprised only 10% of those trial populations. However, the data suggest that survival outcome in metastatic breast cancer is heterogeneous. Some patients suffer from rapidly progressive disease, but a significant number have a more indolent albeit ultimately fatal course [14,15]. Even in the setting of brain metastasis, breast cancer has been shown to be a favorable prognostic factor for survival [16]. It was the impression of the study investigators that patients with breast cancer treated with radiosurgery experienced relatively long survival times compared to other types of tumors. In an effort to quantify this impression we performed a single institution retrospective review of breast cancer patients treated with radiosurgery. Our goal was to determine the influence of known and suspected prognostic factors on survival and to characterize the survival function in our patient population.

Prior retrospective studies have addressed outcomes and prognostic factors for overall survival in patients treated with GKS for breast cancer brain metastasis [17]. Kondziolka *et al.* reviewed outcomes for a cohort of 350 consecutive patients [18]. In this group, 64.9% had undergone previous WBRT and 67% had multiple lesions, with 22% having ≥ 5 metastatic lesions treated. Median survival was 11.2 months, and similar to our results, this study also showed a better overall survival in breast cancer brain metastasis patients compared to historical series looking at brain metastasis without respect to tumor histology. In this study, controlled extracranial disease, KPS ≥ 70, lower total radiosurgery volume, and HER2/*neu* overexpression were all predictors of better overall survival on multivariate analysis. Kased *et al.* reviewed 176 patients with newly diagnosed or recurrent breast cancer brain metastasis [19]. The median survival for new diagnosis treated with SRS was 16.0 months and 11.7 months for recurrent tumors. Overall survival predictors that were found to be significant on multivariate analysis were KPS ≥ 70, estrogen receptor positive tumors, and HER2/neu overexpression. Muacevic *et al.* reviewed 151 patients and determined survival based on RPA status [20]. Median survivals of 34.9, 9.1, and 7.9 months were found for RPA classes I, II, and III, respectively. KPS ≥ 70 and RPA class I were predictors of survival on multivariate analysis. Goyal *et al.* reviewed 43 patients and found a median survival of 13 months [21]. Predictors for increased overall survival in this cohort were found to be higher KPS score, patients with a SIR index (Score Index for Radiosurgery) ≥ 8, and a single lesion.

As in our study, there have been several studies noting an association with improved survival in patients with HER2 overexpressing tumors with CNS disease. *Lower et al.* first demonstrated this finding, and studies since have suggested that the improved survival data was based on HER2-targeted systemic therapies [22-24]. Though HER2 overexpression historically portended a poor prognosis with early distant dissemination of disease, in modern therapy it is believed to provide additional armamentarium for enhanced systemic therapy which has conferred a survival advantage.

In our cohort of patients, we have a rather well distributed population in terms of age and stage, but the patients selected for treatment were uniformly of high performance status (KPS ≥70). While stage had no impact on CNS recurrence rates, there was an association between stage and overall survival. Notably, patients with stage I disease had a shorter MST, as compared to other disease stages. Stage I patients may be less likely to undergo brain surveillance, and if they do develop metastases to the brain, this may implicate relatively more aggressive tumor biology.

The median time to brain failure was 7.1 months with a 1-yr progression-free survival (PFS) of 49% in the brain. In prior reports, 1-yr PFS has varied from 71% to 90% [18,19]. Furthermore the presence of CNS failure was associated with increased survival in our patient cohort. However our report had a very high percentage (46%) of censored cases, as patients who had progressive disease outside of the CNS generally did not undergo further surveillance CNS imaging. This may partially account for the discrepancy in outcomes, as there may be a bias in favor of selection of patients with relatively stable systemic disease or who may be only “slowly” progressing in the brain.

The median overall survival time following GKS for our patient cohort was 12.3 months. Reported times have ranged from 7.8 – 20 months with improved median survival times based on various prognostic assessments [18,20,21,25-29]. In our study, there were no patients with an RTOG RPA of 3, and no reasonable determination could be made regarding trends in survival between RTOG RPA Groups 1 and 2. *Sperduto et al.* presented an updated recursive partitioning analysis for brain metastases, the Graded Prognostic Index in 2008, and subsequently built an enhanced, BS-GPA, with findings reported in 2010 and 2011 [5,30]. In their most recent article, they reported MSTs of 3.4 months for BS-GPA 0.0 - 1.0, 7.7 months for BS-GPA 1.5 – 2.0, 15.1 months for BS-GPA 2.5 – 3.0 and 25.3 months for BS-GPA 3.5 – 4.0. In our cohort, patients with a BS-GPA of 0 – 1.0 had a MST of 6.4 months (likely improved from the *Sperduto et al.* report, as there were no patients in our cohort with a score of 0) [5]. For the BS-GPA of 1.5 - 2.0 the MST was 7.9 months, for 2.5 - 3.0 the MST was 13.3 months, and those with a BS-GPA of 3.5 - 4.0 had a MST of 21.1 months. These findings were generally very similar to those reported in *Sperduto et al.*[5].

While there was no significance to the stratification of patient outcomes based on treatment type, likely due to this analysis being underpowered to detect this difference, there was a trend toward significance with the GKS as a boost after surgery or as salvage after surgery being associated with longer survival times as compared to the other treatment types. Additional analysis in the retrospective setting is fraught with bias. The patients that undergo surgery are a highly selective lot, usually chosen, because of promising features of their disease process. For example, patients who present with delayed metachronous metastases are more likely to undergo resection than patients developing metastases shortly after disease presentation. In addition, there is bias with regards to number of lesions, patient age, patient performance status, etc. We also note a curious outcome in our patient cohort with regards to patients who received upfront WBRT having worse outcomes than those who do not. Once again, there is bias here, in that patients selected for upfront GKS often have a bias towards fewer lesions, better KPS, younger age, and better-controlled extracranial disease.

In conclusion, the strongest individual clinical prognosticators in our cohort were the number of lesions and age, when entered in our stepwise multivariate regression model. While tumor subtype has been demonstrated to be important in other reports, it did not hold in our studies. In addition, performance status did not have an impact on patient survival in our studies. However, this more than likely represents a selection bias in favor of high performing patients within our institution.

## Competing interests

The authors declare that no actual or potential conflicts of interest exist.

## Authors’ contributions

JJ and DF analyzed data and drafted the manuscript. TD performed the statistical analyses. KR, MC, JD, DM, RD, and JS reviewed and edited the manuscript. WM and AP collected patient data. IZ conceived the study, analyzed data, drafted, reviewed and edited the manuscript. All authors reviewed and approved the final draft of the manuscript.
